# RNA-Seq Assembly – Are We There Yet?

**DOI:** 10.3389/fpls.2012.00220

**Published:** 2012-09-25

**Authors:** Simon Schliesky, Udo Gowik, Andreas P. M. Weber, Andrea Bräutigam

**Affiliations:** ^1^Center of Excellence on Plant Sciences (CEPLAS), Institute for Plant Biochemistry, Heinrich Heine UniversityDüsseldorf, Germany; ^2^Center of Excellence on Plant Sciences (CEPLAS), Institute for Plant Developmental and Molecular Biology, Heinrich Heine UniversityDüsseldorf, Germany

**Keywords:** RNA-seq, assembly, plant, NGS, next generation sequencing, transcriptome

## Abstract

Transcriptomic sequence resources represent invaluable assets for research, in particular for non-model species without a sequenced genome. To date, the Next Generation Sequencing technologies 454/Roche and Illumina have been used to generate transcriptome sequence databases by mRNA-Seq for more than fifty different plant species. While some of the databases were successfully used for downstream applications, such as proteomics, the assembly parameters indicate that the assemblies do not yet accurately reflect the *actual* plant transcriptomes. Two different assembly strategies have been used, overlap consensus based assemblers for long reads and Eulerian path/de Bruijn graph assembler for short reads. In this review, we discuss the challenges and solutions to the transcriptome assembly problem. A list of quality control parameters and the necessary scripts to produce them are provided.

## Introduction

Access to a sequence database for a plant species of interest tremendously advances that plant species’ potential use in research, as is evidenced by the success story of the small weed *Arabidopsis thaliana*. However, the complexities of many plants’ genomes and prohibitive costs have precluded the sequencing of their genomes. Instead of the genome, the transcriptomes of tissues of interest for many important crop plants were sequenced^1^. The majority of those sequencing efforts were carried out with substantial funding and frequently in consortia. The advent of next generation sequencing (NGS) technologies has however marked a new era of transcriptomics (Metzker, 2010). Single laboratories are now enabled to produce a sequence resource for their species of choice, be it for commercial, medicinal, ecological, or any other reason. Since the initial proof of concept through the sequencing of the transcriptome of *Arabidopsis* seedlings (Weber et al., 2007), at least 60 additional plant transcriptomes have been sequenced *de novo*. Currently, the 1KP project aims for transcriptomic sequencing of 1,000 plant species^2^.

The quest for a $1,000 human genome has driven the sequencing industries to formidable innovations. The gold rush started with the 454 platform (later acquired by Roche) and the 100 bases long reads that could be obtained on the initial GS20 instrument. Improvements to the platform lead to reads of 250 bases in length. The latest 454/Roche platform used for (plant) transcriptome sequencing is the GS FLX Titanium which allows read lengths of 400 bases (Glenn, 2011)^3^. While a typical 454/Roche sequencing run is finished within less than a day, it yields only 400 Mb per run. Illumina (formerly Solexa) employs a different technology platform. Initially reads were as short as 36 bases but improvements to the technology have led to increased read length of 100 bases (and if paired reads are used, 200 bases of the same transcript). In contrast to the 454/Roche platform, sequencing runs take from several days to more than 1 week but produce ∼600 Gb per run (Glenn, 2011)^4^. With respect to cost per base sequenced, Illumina will beat Roche/454 by a factor of more than 100. Both the 454/Roche and the Illumina platform have been used for transcriptome sequencing and assembly (Table 1). To our knowledge, the two other established NGS technologies, SOLiD and Ion Torrent, have not been used for published plant transcriptome projects (using the search words of RNA-seq, plant AND transcriptome, plant AND NGS at ISI Web of Knowledge).

**Table 1:** **Plant transcriptome sequencing projects until today (complete table available as Table S1 in Supplementary Material)**.

Reference		Plant	Type of reads
Weber et al. (2007)		*Arabidopsis thaliana*	454
Novaes et al. (2008)		*Eucalyptus grandis*	454
Barakat et al. (2009)		*Castanea dentata*, *C. mollissima*	454
Alagna et al. (2009)		*Olea europaea*	454
Dassanayake et al. (2009)		*Heritiera littoralis*, *Rhizophora* mangle	454
Wang et al. (2009)		*Artemisia annua*	454
Swarbreck et al. (2011)		*Avena barbata*	454
Guo et al. (2010)		*Cucumis sativus*	454
Riggins et al. (2010)		*Amaranthus uberculatus*	454
King et al. (2011)		*Jatropha curcas*	454
Hiremath et al. (2011)		*Cicer arietinum*	454
Troncoso-Ponce et al. (2011)		*Ricinus communis*, *Brassica napus*, *Eunonymus alatus*, *Tropaeolum majus*	454
Bräutigam et al. (2011a)		*Cleome gynandra*, *C. spinosa*	454
Cantu et al. (2011)		*Triticum aestivum*	454
Dai et al. (2011)		*Cucumis melo* (sweet melon)	454
Sun et al. (2011)		*Pinus sylvestris*	454
Der et al. (2011)		*Pteridium aquilinium*	454
Franssen et al. (2011)		*Pisum sativum*	454
Ibarra-Laclette et al. (2011)		*Utricularia gibba*	454
Su et al. (2011)		*Phalaenopsis aphrodite*	454
Pont et al. (2011)		*Triticum aestivum*	454
Bleeker et al. (2011)		*Solanum lycopersicum*, *S. habrochaites*	454
Blavet et al. (2011)		Eight *Silene* sp. and *Dianthus*	454
Villar et al. (2011)		*Eucalyptus*	454
Kaur et al. (2011)		*Lens culinaris*	454
Kalavacharla et al. (2011)		*Phaseolus vulgaris*	454
Lu et al. (2012)		*Capsicum annuum*	454
Meyer et al. (2012)		*Panicum hallii* var. filipes	454
Edwards et al. (2012)		*Ziziphus celata*	454
Desgagne-Penix et al. (2012)		*Papaver somniferum*	454
Angeloni et al. (2011)		*Scabiosa columbaria*	454 and Illumina
Garg et al. (2011)		*Cicer arietinum*	454 and Illumina
Krishnan et al. (2011)		*Azadirachta indica*	Illumina
Mutasa-Göttgens et al. (2012)		*Beta vulgaris*	Illumina
Gruenheit et al. (2012)		*Pachycladon fastigiatum, P. cheesemanii*	Illumina and Illumina paired end
Mizrachi et al. (2010)		*Eucalyptusgrandis × E. urophylla*	Illumina paired
Barrero et al. (2011)		*Euphorbia fischeriana*	Illumina paired
Xia et al. (2011)		*Hevea brasiliensis*	Illumina paired
Chibalina and Filatov (2011)		*Silene latifolia*	Illumina paired
Hao et al. (2011)		*Taxus marei*	Illumina paired
Tang et al. (2011)		*Siraitia grosvenorii*	Illumina paired
Wong et al. (2011)		*Acacia auriculiformis, A. mangium*	Illumina paired
Shi et al. (2011)		*Camellia sinensis*	Illumina paired
Hyun et al. (2012)		*Momordica cochinchensis*	Illumina paired
Hao et al. (2012)		*Polygonum cuspidatum*	Illumina paired
Huang et al. (2012)		*Millettia pinnata*,	Illumina paired
Gahlan et al. (2012)		*Picrorhiza kurrooa*	Illumina paired
Zhang et al. (2012)		*Arachis hypogaea*	Illumina paired
McKain et al. (2012)		Different Agavoideae	Illumina paired

## Transcriptome Sequencing and Its Applications

The initial *de novo* plant transcriptome sequencing by mRNA-Seq was conducted on *Arabidopsis thaliana* (Weber et al., 2007). Only half a million reads of close to 100 bases in length were sequenced in this proof of concept approach. It was recognized already at this early stage that remapping the reads to the *Arabidopsis* genome tagged many more transcripts than could be assembled with Newbler, Phrap, or CAP3 (Emrich et al., 2007; Weber et al., 2007). Indeed, assembly was recognized as a future challenge.

Virtually all of the 454/Roche transcriptome sequencing projects following this initial work did have the generation of a transcriptome resource as one of their major objectives (Table 1). Many NGS experiments provide a resource of markers for molecular breeding, for example for eucalyptus, melon, and different legumes (Novaes et al., 2008; Guo et al., 2010; Blavet et al., 2011; Hiremath et al., 2011; Kaur et al., 2011). Other major targets are primary (Dai et al., 2011; Franssen et al., 2011; King et al., 2011; Troncoso-Ponce et al., 2011) and secondary (Alagna et al., 2009; Wang et al., 2009; Bleeker et al., 2011; Desgagne-Penix et al., 2012) metabolism. Plants such as poppy for opium and other alkaloids, tomato for beneficial terpenoids, and Artemisia for artemisinin have been targeted by transcriptome sequencing (Table 1). Adaptations to biotic (Barakat et al., 2009; Sun et al., 2011) and abiotic stress (Dassanayake et al., 2009; Villar et al., 2011) were studied in plants. Finally, transcriptomes of plants carrying a trait of interest such as C_4_ photosynthesis (Bräutigam et al., 2011a; Gowik et al., 2011), weedy habitus (Riggins et al., 2010), being an orchid (Su et al., 2011), a carnivorous plant (Ibarra-Laclette et al., 2011), an ecological model (Blavet et al., 2011), a traditional biochemical model (Franssen et al., 2011), or an endangered species (Edwards et al., 2012), were analyzed. Since 454/Roche pyrosequencing was used, the number of sequenced reads is comparatively low, between 0.08 and 3.3 million reads (Table 1). The majority of the assemblies were realized with overlap consensus based assemblers such as CAP3 (Huang and Madan, 1999; four instances) or its implementation in the clustering pipeline TGICL (Pertea et al., 2003; five instances), which prefaces CAP3 with a megablast to reduce the number of sequences fed to CAP3 and hence RAM requirement. MIRA (Chevreux et al., 2004; one instance) and one of the multiple Newbler versions^5^ (seven instances) were also frequently used. In four projects a combination of two assemblers was used. CLC^6^, LEADS (Dai et al., 2011), Paracelsus Transcript Assembler (Novaes et al., 2008) and Seqman Ngen (Edwards et al., 2012) were each used in a single published assembly (Table 1). The different assemblies were quality controlled – if they were controlled at all – by different parameters. Hence it is difficult to compare the different assembly methods. All assemblies report the number of unigenes (the sum of assembled contigs and unassembled singletons) and either the N50 or the average length of the contigs. These two parameters can be compared with reference sequence numbers, average sizes and N50 from predicted transcriptomes of species with sequenced genomes. The parameters show that the assemblies are far from perfect and that none of the assemblers achieves a satisfactory reconstruction of an actual transcriptome. While the representation of the transcriptome was the expressed goal of these studies, none of them fully succeeded. Most of the assemblies were carried out either with Roche’s Newbler or with a decades-old tool, CAP3. No marked improvements could be detected in the assembly parameters unigene number and average length over time (Table S1 in Supplementary Material).

Although one may be tempted to dismiss such error prone, incomplete assemblies, the majority of them have already proven themselves useful for downstream applications such as proteomics (Bräutigam et al., 2008; Franssen et al., 2011) or pathway reconstruction (Wang et al., 2009; Bräutigam et al., 2011a; Dai et al., 2011; Troncoso-Ponce et al., 2011; Desgagne-Penix et al., 2012). The databases were developed to provide a sequence resource for future experiments. The analysis of single genes involved in the C_4_ photosynthetic pathway based on hypotheses derived from RNA-seq experiments has already been successful (Furumoto et al., 2011; Sommer et al., 2012). Hence even imperfect assemblies succeed in enabling future research. Downstream approaches that require perfect or near perfect unigenes such as the evolutionary analysis of gene family expansions will likely suffer more from the current shortcomings of these assemblies.

RNA-seq by Illumina sequencing was initially used for transcriptome sequencing in species with sequenced genomes (e.g., Vega-Arreguin et al., 2009; Li et al., 2011). It has been successfully applied to produce transcriptomes *de novo* (Table S1 in Supplementary Material). The technology appeals to researchers despite its comparatively short reads because it produces much larger coverage at the same or a lower price. However, it presents a new set of challenges for the assembly.

Similar to 454/Roche based sequencing projects, virtually all Illumina based RNA-seq experiments on non-model species have been conducted to produce a transcriptome database. RNA-seq using the Illumina technology was undertaken to analyze transcriptomes for plants of nutritional or medical value (Barrero et al., 2011; Hao et al., 2011, 2012; Krishnan et al., 2011; Tang et al., 2011; Gahlan et al., 2012; Hyun et al., 2012) or of commercial value (Mizrachi et al., 2010; Shi et al., 2011; Xia et al., 2011; Mutasa-Göttgens et al., 2012; Zhang et al., 2012). Two experiments addressed ecological and evolutionary questions, the evolution of sex chromosomes (Bergero and Charlesworth, 2011) and the phylogenetic positioning of species (McKain et al., 2012). The majority of sequences were produced with paired end technology. In this case, sequences from both ends of fragments of defined size are sequenced. The use of paired ends allows scaffolding: sequence reads are used to produce contigs. The information which reads belong together and their specific distance orders disconnected contigs on scaffolds. The unknown nucleotides in the gaps of scaffolds are caused by knowing the size of the gap but not the identity of the nucleotides and hence the nucleotides in the gap are denoted as Ns. One assembler that was originally developed for genome assemblies, SOAPdevono^7^, has been used to assemble the majority of plant transcriptomes. Additional assemblers used include CLC, velvet (Zerbino and Birney, 2008)^8^, AbySS (Simpson et al., 2009), and Trinity (Grabherr et al., 2011). In one of the projects a custom resolution algorithm for velvet was developed and used (Mizrachi et al., 2010; Table 1). This customized velvet version has produced the best assembly in terms of contig number and average contig length. Despite its success, the method has not been used for any of the other projects.

Finally, RNA-seq experiments have combined both 454/Roche and Illumina sequencing. Transcriptomes of chickpea and pincushion flower were produced using both technologies and hybrid assemblies (Angeloni et al., 2011; Garg et al., 2011). Although promising in prospect of complementary error correction, to date, true hybrid assembly approaches are limited to an assembly of one library (often 454) as a base transcriptome and subsequent correction of the consensus sequence by mapping the other read library (Illumina or SOLiD). Quality improvements of transcriptome hybrid assemblies have not yet been assessed in a comparative study. However, in the context of genome assembly it was shown that a stepwise (as explained above) hybrid assembly had a higher quality (according to the authors: comparable to Sanger-sequencing) than single library approaches (Aury et al., 2008). The use as well as the strategy of hybrid assemblies is currently vigorously discussed in the online community ^9^^,^^10^.

Overall, similar to the assemblies from 454/Roche RNA-seq experiments, those from Illumina technology suffer from limitations. It will be crucial to continue developing assemblers with enhanced capability while establishing standard quality controls to make assemblies from different species, technologies, and assembly strategies comparable.

## Assemblers

Two principally different types of assemblers are available for RNA-seq data: overlap-layout-consensus (OLC) assemblers and Eulerian path assemblers which are based on de Bruijn graphs (summarized in Flicek and Birney, 2009).

Overlap-layout-consensus assemblers were developed for Sanger sequences. In principle, the assembler starts with a sequence read, looks at its sequence, and searches the read space for another read that contains an overlapping sequence. The overlap is specified by its length and the number or percentage of matching bases. The memory requirement for this operation depends on the number of reads to be searched. Thus, more reads require more computer power. Already during times of Sanger-sequencing, this method became inefficient with the available computers and a prefacing clustering step was added. This clustering step groups sequences deemed similar, for example by a megablast search (Pertea et al., 2003). The assembler then only searches the sequences in each cluster. The three most prominent examples for these OLC based assemblers are Newbler (Roche/454 Life Sciences, Branford, CT, USA), MIRA (Chevreux et al., 2004), and CAP3 (Huang and Madan, 1999; or TGICL which uses megablast and CAP3). While these assemblers are suitable for 454/Roche sequences, the number of reads generated with Illumina are simply too large to be processed. In an assessment of different assemblers with both simulated and real data, TGICL was superior to MIRA and CAP3 in its results (Bräutigam et al., 2011b). No new assemblers have been developed and used except for Newbler developed by the company 454/Roche itself.

**Figure 1:**
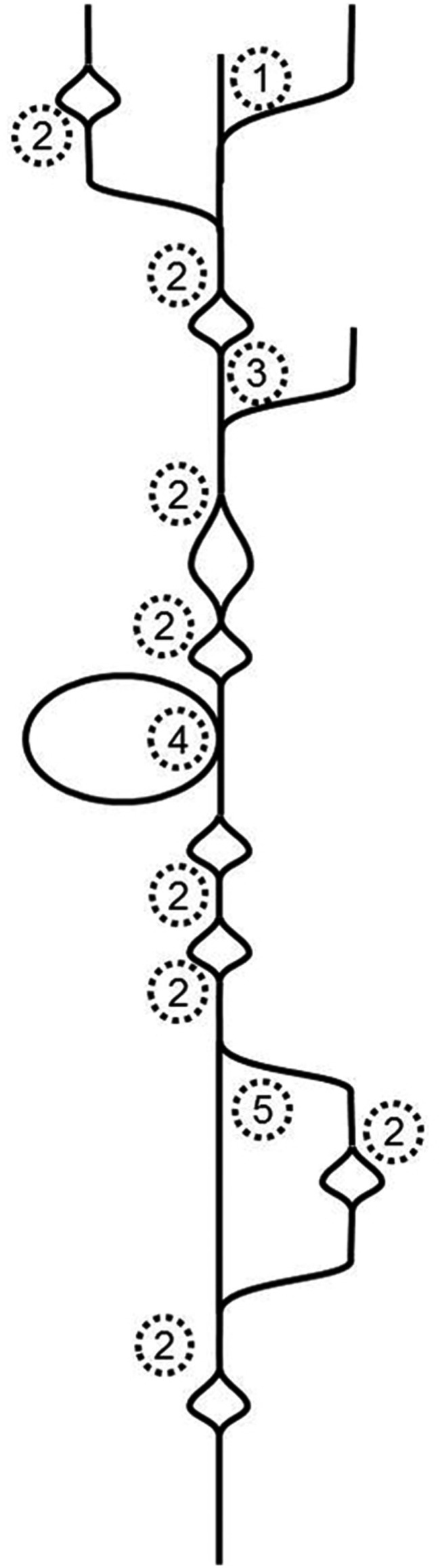
**Schematic de Bruijn graph of a single transcript; 1 alternative transcription start site *or* hybrid joining *or* DNA contamination; 2 SND caused by a sequencing error *or* a SNP *or* mutation after gene duplication; 3 alternative transcription start site *or* DNA contamination; 4 alternative exon use; 5 alternative exon use *or* mutations after recent gene duplication**.

**Figure 2:**
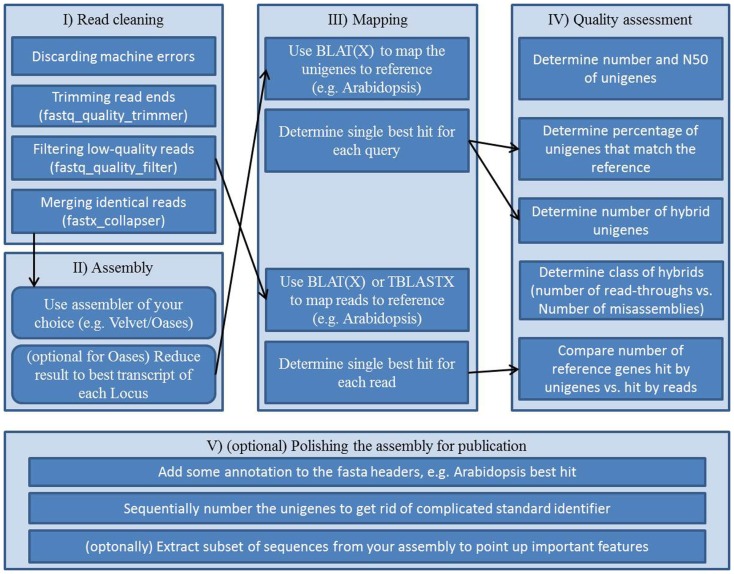
**Workflow scheme for a transcriptome assembly and quality assessment: (I) preprocessing of the raw reads, (II) assembly of processed reads, (III) mappings for annotation and for subsequent quality assessment, (IV) collecting quality information from assembly and mappings, (V) final polishing to create an easy to use, thus easy to share file from the assembly**.

To tackle Illumina-generated sequence reads, a new type of assembler was created. It is based on finding the Eulerian path through a de Bruijn graph (Pevzner et al., 2001). Essentially, this type of assembler breaks the whole sequence space in pieces of defined length, which are called k-mers. It then moves along the k-mers and creates a graph in the process. Identical overlaps of k-mers are merged and counted. If the assembler encounters differences, the graph will branch, if it subsequently encounters identity again, the graph will join the ends. That means that single nucleotide differences (SNDs) will produce bubbles (Figure 1; 2). Such SNDs can either represent a sequencing error or genetic variation in form of a single nucleotide polymorphism (SNP). Large bubbles and open ended branches can be caused by alternative splicing and alternative transcriptional starts and stops (Figure 1; 1). The presence of genomic DNA in the sample, improperly trimmed and filtered reads, sequencing errors, alternative splicing, and background transcription will lead to many more deviations from the one transcript, which ideally should look like a straight line. In reality the graph has no straight lines but is full of bubbles and frayed ends (Figure 1). When such a graph is resolved, the researcher wants all “real differences” such as alternative splicing events, transcripts resulting from recently duplicated but still very similar genes, and genetic variation, for example from different alleles of a particular genetic locus, represented. However, all differences caused by technical errors should be removed. The only information available for the algorithm to resolve the graph is the number of instances observed for each k-mer. If such a graph is used for genome sequencing of organisms without complex genomes (i.e., not plants), the application for which it was developed, the graph can be resolved using the degree of coverage for each k-mer. In theory, the number of reads that cover each base in the graph should be equal for the whole graph. While this does not hold true for repetitive sequence elements, it can be used to resolve the remainder. Given 100-fold coverage in a genome homozygous at all loci, you would require that each k-mer is covered at least, say, 80 times to be called real. If the coverage is lower, it is likely a sequencing error.

The resolution of transcriptome graphs is very different from the resolution of genome graphs. The dynamic range of a leaf transcriptome spans at least five orders of magnitude (Bräutigam et al., 2011a; Gowik et al., 2011). Hence the coverage of a transcriptome is the polar opposite of even. SNPs and InDels present in natural populations cause uneven coverage. Transcripts with higher diversity in the population exhibit more changes (as represented by bubbles in Figure 1) than transcripts with lower diversity in the population. Alternative splicing and start and stop sites will cause differential coverage. If an exon is only used 10% of the time, it may not make it past the resolution cut-off.

To solve the problem of uneven coverage, the assemblers that were originally designed to produce genomic assemblies, such as ABySS, SOAPdenovo, or velvet, have been extended with add-ons for the assembly of transcriptomes, such as Trans-ABySS, SOAPdenovo-Trans, or velvet/Oases. Even given this amendment, assemblers do not succeed in assembly as evidenced by contig numbers that are much higher than the expected transcript number and average contig sizes much lower than that of an average transcriptome (Table S1 in Supplementary Material). Assemblers for short reads remain limited and both the development of new assemblers as well as post-assembly processing and parameter optimization is ongoing. The detection of genetic variation and transcript variants will likely require post-assembly read mapping and evaluation through the researcher.

## Considerations for NGS Transcriptome Assembly

The key differences between NGS and Sanger sequence reads are the number of reads and the length of the reads. Even using the long-read technology 454/Roche, the reads are only half to a third as long as compared to Sanger sequences. With a single NGS run, half a Gigabase to several Gigabases of sequence data is generated. In consequence, the challenge has shifted from efficiently generating sequence reads to efficiently assembling them. Given an error rate of ∼1% and 40,000 reads of 400 bases length for a gene of 1 kb, 160,000 incorrect base calls are expected. If these are randomly distributed, on average, each single base will be called incorrectly about 160 times. Even assuming error rates of only 0.1%, each base will still be called incorrectly 16 times. For this reason, there is a correlation between the number of contigs resulting from a transcript and the expression strength of the corresponding gene (Franssen et al., 2011). The large number of sequencing reads calls for intense sequence pruning. There are several software packages that include pruning pipelines, such as the fastx-toolkit^11^, the fastQC software^12^, and the RobiNA package (Lohse et al., 2012). Those are used to determine average quality per base in addition to other quality control parameters. Reads can be trimmed (pruned at the ends if bases are below a quality threshold), filtered (if internal bases are below a threshold), and purged from duplicates (merging multiple, identical reads into a single sequence). Unfortunately, the majority of assembly publications do not report their pruning pipeline and threshold values; they restrict themselves to stating the number of high quality bases that were fed into the assembly pipeline.

In theory, the error problem was solved if one were to assemble only reads with a high coverage cut-off during the graph resolution. In that case, sequencing errors were ignored because their k-mer numbers are too low. However, due to the large dynamic range of the transcriptome, low abundance genes, such as transcription factors and regulatory kinases, are underrepresented (Czechowski et al., 2004). These genes are discriminated against if the assembly is processed with high coverage cut-offs during resolution (Schliesky and Bräutigam, unpublished observations). They simply disappear. Similarly, rare transcript isoforms will also be discarded during the resolution step if high coverage is required.

Library normalization at least partially addresses the challenge of a high dynamic range. Normalization by digestion reduces the dynamic range by one order of magnitude (Christodoulou et al., 2001) but normalized libraries clearly retain some dynamic range (Franssen et al., 2011). While normalization likely improves the assembly, it comes at a cost: sequence information and quantitative information are no longer collected at the same time. If quantitative information is not required, normalization is highly recommended.

At least low coverage transcripts could be recovered if one knew before assembling how many reads are produced from each transcript and adjust the resolution algorithm accordingly for each piece of the graph. Possibly, a dynamic approach – assembly, read mapping on the preliminary assembly, re-assembly with sliding scale of resolution coverage cut-off – might be able to solve the problem. While none of the current transcriptome assemblers has implemented this strategy, its application for one Illumina plant transcriptome assembly may serve as the proof of concept for the approach (Mizrachi et al., 2010).

The key challenge in assembly is weeding out all variation caused by sequencing errors, library preparation, and other technical artifacts while keeping all variation caused by biological phenomena such as genetic variation, alternative splicing, and others.

## Assessing the Assembly

In principle, assessing an assembly is easy – it should accurately reflect the transcriptome of the sequenced tissue and species. In practice, the accurate transcriptome is unknown and not available for comparison. Two different approaches to overcome this problem can be envisioned. (i) Establishing assembly parameters with simulated reads from a reference species and transferring those to *de novo* sequencing and (ii) assembling *de novo* transcriptome and estimating reference parameters. While the first possibility has immediate appeal, there are a number of obstacles. The dynamic range of transcriptomes is different in different tissues and between species (Fluhr et al., 1986). A method optimized for a root transcriptome might not necessarily work well with a leaf transcriptome and *vice versa*. Different read length, paired end or single end sequencing, or different sequencing depth dictated by the available instrumentation and funding will likely change the parameters for the best possible assembly. Carrying out the optimization with a non-target dataset will also cause substantial time investment with little return in the beginning since not even a working assembly of the target transcriptome is created. For all these and possibly additional reasons, many researches will immediately start to work on the target transcriptome. If a common set of assessment parameters were developed, all possible transcriptomes could be measured against these parameters and thus compared with each other.

### Number of unigenes

The number of unigenes expected from an assembly can be calculated with a Fermi estimate. The gene number for the majority of sequenced plant genomes is between 20,000 and 40,000. Using microarray data from *Arabidopsis*, one can estimate that about one half of the genes are expressed in leaves. Using these two numbers as approximations, the Fermi estimate for loci expected from a leaf transcriptome is about 15,000. While species with a very recently duplicated genome may have close to twice as many, none will have an order of magnitude more transcripts (compare to Table S1 in Supplementary Material). However, the number of unigenes can be easily manipulated while not gaining a better assembly. One strategy crops the unigenes by a minimal-length cut-off. While it facilitates subsequent read mappings it severely discriminates against “real,” short transcripts. Another example, raising the coverage cut-off during graph resolution will reduce the number of unigenes. This strategy indeed removes unigenes constructed because of sequencing errors but it will also discriminate against low abundance transcripts as discussed above. It is thus important to combine these measures with the number of reference transcripts matching the unigenes.

### Number of reference transcripts matching the unigenes

Once the assembly is complete, it needs to be compared to the most closely related reference species. The unigenes are matched to the reference sequence by Blast or Blat (Kent, 2002). While it is unknown how many reference transcripts should be tagged by the assembled unigenes, a higher number of tagged references indicate a more inclusive and thus better assembly. Genes that are not expressed will never be tagged but as long as the number of tagged genes increases during assembly optimization, the assembly is getting better in terms of inclusiveness.

### Number of reference transcripts hit by reads compared to number of reference transcripts hit by unigenes

It is possible to estimate the number of unigenes produced by the assembly. If the reads are at least 75 bases long after trimming and filtering, they can be mapped to a reference transcriptome provided that the reference species is reasonably closely related. However, in reality “reasonably close” will not be sufficient to produce a perfect mapping. Therefore (i) a traditional mapping program that allows for multiple mismatches (i.e., BLAST or BLAT) and (ii) mapping in protein-space (i.e., translated query against translated database; blatx or tblastx) improves the mapping success with respect to evolutionary distance. In theory, reference transcripts tagged by reads are expected to be tagged by unigenes. This assumption is only true if a loss-less assembler such as OLC assemblers are used. Reads that do not overlap with other reads are reported as singlets or singletons when using these assemblers. The resolution cut-off applied in graph-based assemblies will overlook unigenes if they are not covered by at least the coverage cut-off. Mapping reads to a reference results in estimated read numbers per locus. With these read numbers one can check how many reads are actually needed to produce a contig or a full length contig based on different assembly parameters such as k-mer size and coverage cut-off. Surprisingly, the assembly will also produce unigenes for which no read tagging was recorded. In that case, the setting of either Blat or Blast was too stringent too match the reads but the longer unigene produces a match. This quality control measure will overlook lineage specific transcripts that have no match in the reference transcriptome. While every genome sequencing approach does reveal lineage specific genes, the number of genes present in multiple plant lineages is vastly higher.

The ratio between reference sequences tagged by reads and those tagged by unigenes should ideally approach 1:1.

### N50, average length, median length

These three parameters are always reported with genome assemblies. The N50 can be envisioned as follows: if you order the unigenes by their length and then start counting nucleotides at the largest unigene, the N50 will report the unigene length at which you have counted through half of the bases. While this is a sensible measure for genomes, it makes less sense for transcriptomes. After all, with genomes you expect as many contigs as you have chromosomes. In transcriptomes, you may have different N50s for different tissues of the same plant since different groups of genes are expressed. The same caveat is true for the average length and the median length.

While different (whole) transcriptomes indeed have slightly different parameters with regard to N50, average length and median length, the values are similar enough to yield an estimate for the expected values for an unknown transcriptome (compare to Tables 1 and 2).

**Table 2:** **Quality assessment parameters drawn from transcripts of publicly available genome databases**.

Species	Genome size (Mbases)	Number of transcripts including isoforms	N50	GC%
*Arabidopsis thaliana*	120	41671	1912	42.27
*Brassica rapa*	485	41019	1482	46.28
*Populus trichocarpa*	481	45033	1845	42.29
*Solanum lycopersicum*	950	35802	1461	41.61
*Oryza sativa*	420	66338	2295	51.30
*Setaria italica*	515	40599	1811	52.75
*Zea mays*	2066	136770	1612	51.14

### Length of the longest unigene

The length of the longest unigene might not represent a sensible measure. If the sequencing library was contaminated by genomic DNA, a large fraction of this DNA will come from the plastid genome. The plastome DNA is known to be AT-rich and thus survives the poly-A enrichment step during the Illumina mRNA enrichment protocol well (Schliesky, Mullick and Bräutigam et al., unpublished observations). Its presence leads to remarkably long contigs in the assembly albeit not quite to an assembly accurately representing the transcriptome. A second consequence of DNA contamination is the presence of many contigs matching transposon-like sequences which are also AT-rich. The complete or near complete presence of a unigene matching the longest nuclear transcript of a reference also only shows that the assembly parameters were ideal for that transcript but not for all transcripts in the sequenced library.

### Number of estimated full length unigenes

While the length of the longest unigene may not be an ideal measure, the estimated number of full length unigenes reflects on the success of the assembly. The unigenes are matched to a transcriptome reference from a closely related species. While during evolution, genes will have extended or contracted, on average, their length will remain comparable. More unigenes that reach the length of the reference transcripts indicate a better assembly.

If no reference seems suitably close enough, it is still possible to compare the length distributions qualitatively. Comparing multiple publicly available plant transcriptome databases with respect to their length distributions demonstrates an overall pattern on what a transcriptome should possibly look like (e.g., ∼90% of the sequences between 200 and 3500 nt length). In practice that is not achieved because assembly software often produces a huge fraction of truncated transcripts between 0 and 200 nt length.

### Number of hybrid/read-through unigenes

While full length unigenes are the goal of an assembly, no hybrid unigenes should be produced. These result from the joining of two target transcripts matching two different reference transcripts into one unigene. Two different kinds of hybrid unigenes can be produced. Illumina resequencing of *Arabidopsis* leaf transcriptomes identified unigenes that were assembled from adjacent transcripts (Schliesky, unpublished). Read mapping to the genome revealed that these hybrid unigenes resulted from read-through transcription. They thus likely reflect the true transcriptome. The second class of hybrid unigenes is undesirable. In this case, the similarity of sequences, sequencing errors, or incomplete read trimming and filtering cause the merging of two target transcripts into one reference unigene. A read mapping in this case identifies no evidence for this feature. Different assembly parameters favor or do not favor the creation of this second class of hybrids (Schliesky, unpublished) and thus hybrid detection should be included in the quality control. One strategy for hybrid detection by alignment to *Arabidopsis* could be designed as follows. Based on the outcome of an alignment, all unigenes that map to multiple genes get tagged as hybrid (also known as chimera or fusion genes), if the match takes place in distinct, i.e., non-repetitive, sections of the unigene sequence. Subsequently the chromosomal position is used to classify the type of hybrid to either read-through (matching neighboring genes) or second class hybrids (matching non-neighboring genes). A high proportion of second class hybrids points to a bad assembly algorithm, to bad assembly parameters (e.g., k-mer too large) or to a contamination of some sort (e.g., genomic DNA or low quality reads).

If no closely related reference is available, the hybrid detection strategy probably needs to be amended. With increasing evolutionary diversity mapping accuracy will decrease. Therefore mapping errors may lead to incorrectly detected hybrids. That may be solved by increasing the required matching length during mapping (increasing accuracy) at the cost of not mapping some unigenes at all (decreasing sensitivity). Alternatively, hybrid unigenes may be detected by mapping the reads back to the unigenes. At the position of error, read coverage is likely lower than in the adjacent regions. Detecting and cropping those bridging regions reliably will reduce the number of hybrid transcripts. This approach is based on same idea as an assembly algorithm with a sliding resolution window for per base coverage. If the quality assessment was completely independent of a reference sequence, lineage specific genes which have no match in reference database would also be included in the quality assessment.

## Example Workflow

As a step toward comparable transcriptome assessments a collection of Perl and Unix scripts, which are automating parts of the assessment, is provided in this review. It resembles an example workflow (Figure 2, Supplementary Presentation 1) for assembling and assessing reads of *Arabidopsis* mRNA. This out-of-the-box pipeline consists of five blocks; (i) vigorous read pruning, (ii) assembling, (iii) mapping to a reference, (iv) collecting quality parameters, and (v) polishing the assembly for publication.

Carrying out transcriptome assembly in a standardized way has not been publicly pursued prior to this review. In order to keep the workflow repeatable and comparable we provide a step by step instruction set on how to use the supplemental scripts to assemble a sequencing run and conduct quality assessment on the assembly. Please be aware that the workflow including all scripts was designed with *Arabidopsis* as the target reference. Scripts might or might not be adaptable to other species. The workflow was established and tested on a Linux machine running 64 Bit Ubuntu 10.04 and having installed BioPerl, BioPython, the FASTX-toolkit, BLAT, and BLAST.

First, all scripts need to be extracted and copied into a folder (Supplemental Scripts 02–12), together with the raw reads (fastq.gz files) and the reference. Start a terminal and change to the directory containing the scripts. All commands needed are in Supplemental Script 1. Lines proceeded by a #-symbol present comment lines and are used for explanation. Illumina reads obtained from a sequencing facility are supplied as *.fastq.gz files. To unzip and concatenate them, the zcat command is used (Supplemental Script 1 line 4).

### Read cleaning (supplemental script 1 lines 6–11)

While reads coming off the sequencer are not dirty in the traditional sense, they may contain low quality reads, adaptor sequences, and low quality bases. Reads are cleaned to remove as much non-biological variation as possible. As discussed previously read cleaning is crucial for a good assembly. The workflow starts by removing reads flagged as inappropriate by the sequencer (line 7). For quality trimming knowledge of the average overall base quality is needed. This is evaluated using the FASTX-Toolkit (line 8). Visual aids (e.g., fastq_quality_boxplot_graph.sh) may ease interpretation of the results. The stats file provides one line per base (i.e., in Illumina 101 bp reads 101 lines) and for each base a median quality score is calculated. Frequently, read quality will be low toward the end of the read. If at any point, say from base 86 to base 87, the median quality drops dramatically, the ideal quality cut-off will be in between this range. For sequencing runs with good library preparation and no problems during the sequencing we recommend a cut-off of 30.

The actual cleaning is conducted in three steps; (i) trimming (line 9), which prunes the ends off of the reads if they are below the defined quality cut-off and subsequently discards all reads that are shorter than a defined length cut-off (we suggest half the read length, i.e., 50) after trimming. (ii) Filtering (line 10), which discards all reads that do not meet the required quality cut-off with at least a defined length (in percent of the total read). For the majority of sequencing runs, the values suggested above are a good starting point. Trimming and filtering does not discard more than 15% of the reads if library preparation and sequencing went well. In other cases, values might have to be adjusted and trimming and filtering values might have to be relaxed. (iii) Collapsing (line 11), since memory requirements are lower if fewer reads are assembled.

### Assembly (supplemental script 1 lines 13–27)

Lines 13–27 contain an out-of-the-box pipeline from cleaned reads to assembled best transcript isoforms using Velvet/Oases. The pipeline can be adapted for other assemblers. Velvet/Oases is called in three steps. In the first one, output directory, k-mer size and input files are declared (line 15). In the subsequent steps a de Bruijn Graph is built (line 16) and resolved with an algorithm optimized for transcriptomes, i.e., Oases (line 17). Oases outputs a huge amount of transcripts, which is due to the fact that Oases resolves bubbles and branches in the Graph into all possible transcript isoforms of a locus. The number of transcripts is, compared to the number of unique loci detected by Oases, frequently two (or more) times higher. Picking the best transcript for each locus is a challenge as there is no standard to what “best” means. The longest transcript is often the least supported (i.e., covered by k-mers), whereas the most supported often is the shortest one. To solve this problem a script (line 22, Supplemental Script 02) has recently been published on Google Code^13^ (by Adrian Reich 2012) that essentially chooses the most supported transcript (i.e., highest k-mer coverage) that has at least XX% length of the longest transcript in this locus. In our hands a length cut-off of 20% showed the best results in subsequent quality assessment.

Many assembly papers include a length cut-off to reduce the number of transcripts. Although this curation is, in essence, cheating with the number of unigenes, the pipeline includes a Perl script for cropping the database (line 27, Supplemental Script 03).

### Mapping (supplemental script 1 lines 29–46)

A major bottleneck – conceptually as well as computationally – if working with non-model species is the read mapping. When working on non-model species there is no sequenced genome available to use as a reference. Mapping to a close relative works if precautions are taken to account for the evolutionary distance. Modern mapping algorithms are designed for speed and allow only one mismatch. These algorithms will fail to map to a related reference. Therefore in cross-species mapping the use of traditional mapping algorithms like BLAST and BLAT in protein-space is recommended. While mapping unigenes to the reference (line 31) finishes in the order of minutes, mapping reads to the reference will take much longer (depending on the library size in the order of weeks). This limitation can be bypassed by parallelizing BLAT with a script (line 34–36, Supplemental Script 04) on the number of CPUs available. The script splits the read file, starts parallel single BLAT runs and merges the results. The number of CPUs can be changed within Supplemental Script 04 in line 3 (default is 2). Alternatively BLAST, which natively supports multiple CPUs, can be used for the mapping (line 39, 40).

Multiple mappings can be resolved to only one single best hit per query (i.e., per read) by using the best hit scripts for either BLAST (line 42, Supplemental Script 05) or BLAT (line 46, Supplemental Script 06).

### Quality assessment (supplemental script 1 lines 48–87)

As discussed above the most frequently used measures to evaluate the quality of an assembly are number of unigenes and N50. A Perl script to calculate the read length histogram of a fasta file (line 50, Supplemental Script 07) was developed by Joseph Fass (modified from a script by Brad Sickler). The script produces a histogram that can be easily visualized, and calculates the number of unigenes, N25, N50, and N75.

The percentage of unigenes that match a reference are calculated using the total number of references and the number of matching unigenes. The total number of references is counted (line 54). The number of unigenes which map to a reference is produced by extracting the query identifiers from the mapping table and by counting unique occurrence (line 56). The mapping efficiency (ratio of mappable unigenes by total references) can be interpreted as a measure of completeness with the caveat that single tissue transcriptomes are not expected to represent a complete transcriptome.

Hybrid unigenes can be detected with the help of mapping. In hybrid unigenes, different sections of the unigene map to different loci in *Arabidopsis*. These hybrid unigenes can either be read-throughs of two adjacent genes or misassemblies. While it is desirable to have no hybrid unigenes that represent transcripts fused by the assembler, it might add to the understanding of cellular mechanisms to identify read-throughs. Therefore we provide two Perl scripts, which (i) detect any hybrid unigenes (line 60, Supplemental Script 08) and (ii) subsequently classify those as read-throughs or not (lines 63–67, Supplemental Script 09). While hybrid unigenes are undesirable in an assembly, they can be tolerated for single gene analysis. A read mapping provides visible cues whether coverage is even or whether parts of the unigene are only supported by few reads. Only with more and more transcriptomes being assembled and large scale comparisons enabled, hybrid unigenes will become an issue in comparison.

The quality of an assembly can also be measured by comparing the number of reference genes hit by unigenes with the number of reference genes hit by reads. This is based on the assumption that genes, which are expressed (i.e., hit by a read) will generate a transcript (i.e., unigene) during the assembly which maps to the same reference. Comparing the numbers of genes hit by reads (lines 70, 71) and by unigenes (lines 74, 75) provides a quick assessment whether those values are in the same range. Subsequently, it is assessed whether the reference genes hit by reads are also hit by unigenes. This question is answered using standard Unix commands and set theory. Given two files “genes hit by unigenes” and “genes hit by reads” with a unique set of identifiers in each, adding (i.e., concatenating) one file and twice the other file yields a new set which has each identifier either occurring once, twice, or three times. Extracting lines by count yields three groups, (i) genes only present in the file used once (line 84), (ii) genes only present in the file used twice (line 85) and (iii) genes that are present in both files and therefore commonly hit by unigenes and reads (line 86). A large percentage of the latter group indicates that the assembled transcripts reflect the expressed genes. An alternative way to determine the intersect between two files is based on the Unix “join” command (lines 90–92).

### Final polish of the assembly (supplemental scripts 1 lines 89–99)

Prior to publication, an annotated fasta database of the assembly needs to be generated. The scripts provided incorporate an annotation to the sequence headers, e.g., best hit in *Arabidopsis* (lines 96, 97, Supplemental Script 10) and number the identifiers of unigenes sequentially to get rid of awkward assembler headers (line 100, Supplemental Script 11). If only a subset of sequences are needed a Perl script (line 104, Supplemental Script 12) can extract it if given a one-per-line list of identifiers.

### Applying the workflow (quick and dirty)

The complete workflow discussed in this review is attached as a script (Supplemental Presentation 1) and could be run unsupervised. This requires the fastq.gz files to be in the same folder as all the Supplemental Scripts along with an *Arabidopsis* reference that is named “TAIR10_cdna.fasta”. Additionally Perl, Python, BioPerl, BioPython, BLAST, BLAT, Velvet, Oases, and the FASTX-Toolkit have to be installed on the system. The hardware requirements of the assembly in terms of memory are rather high. Assembly was limited to 50 M reads with 96 GB RAM available.

Due to these strict requirements, we strongly recommend reading and adjusting the workflow to your specific needs. All scripts have either a help output (if ran with - - help or - ? as parameter) or a Perldoc documentation (opened by running “perldoc *script_name*”) or both.

## Conclusion

Next generation sequencing and transcriptome assembly have already proven beneficial for research. However, current assemblies are still far away from an accurate representation of a transcriptome. Detailed description of the assembly method including read treatment prior to assembly, assembly parameters, and stringent quality control will make different assemblies more comparable and will make it easier to reproduce successful assemblies. This first attempt to bring the quality assessment in line helps to make transcriptomic resources much more comparable and reusable for the community. At the very least, each assembly publication should include a fasta file with all unigenes. Until full length single molecule sequencing for transcriptome sequences becomes technically feasible, transcriptome assembly will remain the major bottle neck during transcriptome sequencing. We are not there yet!

## Conflict of Interest Statement

The authors declare that the research was conducted in the absence of any commercial or financial relationships that could be construed as a potential conflict of interest.

## Supplementary Material

The Supplementary Material for this article can be found online at http://www.frontiersin.org/Plant_Systems_Biology/10.3389/fpls.2012.00220/abstract.

## Footnotes

^1^http://compbio.dfci.harvard.edu/tgi/plant.html

^2^http://www.onekp.com

^3^http://www.molecularecologist.com/next-gen-fieldguide/

^4^http://www.molecularecologist.com/next-gen-fieldguide/

^5^http://454.com/products/analysis-software/index.asp

^6^http://www.clcbio.com/

^7^http://soap.genomics.org.cn/soapdenovo.html

^8^http://www.ebi.ac.uk/~zerbino/oases/

^9^http://www.seqanswers.com

^10^http://www.biostars.org

^11^http://hannonlab.cshl.edu/fastx_toolkit/index.html

^12^http://www.bioinformatics.babraham.ac.uk/projects/fastqc/

^13^http://code.google.com/p/oases-to-csv/
